# Type VII Collagen Expression in the Human Vitreoretinal Interface, Corpora Amylacea and Inner Retinal Layers

**DOI:** 10.1371/journal.pone.0145502

**Published:** 2015-12-28

**Authors:** Bart Wullink, Hendri H. Pas, Roelofje J. Van der Worp, Roel Kuijer, Leonoor I. Los

**Affiliations:** 1 Department of Ophthalmology, University Medical Center Groningen, University of Groningen, Groningen, the Netherlands; 2 W.J. Kolff Institute, Graduate School of Medical Sciences, University of Groningen, Groningen, the Netherlands; 3 Department of Dermatology, University Medical Center Groningen, University of Groningen, Groningen, the Netherlands; 4 Department of Biomedical Engineering, University Medical Center Groningen, University of Groningen, Groningen, the Netherlands; Rutgers University, UNITED STATES

## Abstract

Type VII collagen, as a major component of anchoring fibrils found at basement membrane zones, is crucial in anchoring epithelial tissue layers to their underlying stroma. Recently, type VII collagen was discovered in the inner human retina by means of immunohistochemistry, while proteomic investigations demonstrated type VII collagen at the vitreoretinal interface of chicken. Because of its potential anchoring function at the vitreoretinal interface, we further assessed the presence of type VII collagen at this site. We evaluated the vitreoretinal interface of human donor eyes by means of immunohistochemistry, confocal microscopy, immunoelectron microscopy, and Western blotting. Firstly, type VII collagen was detected alongside vitreous fibers6 at the vitreoretinal interface. Because of its known anchoring function, it is likely that type VII collagen is involved in vitreoretinal attachment. Secondly, type VII collagen was found within cytoplasmic vesicles of inner retinal cells. These cells resided most frequently in the ganglion cell layer and inner plexiform layer. Thirdly, type VII collagen was found in astrocytic cytoplasmic inclusions, known as corpora amylacea. The intraretinal presence of type VII collagen was confirmed by Western blotting of homogenized retinal preparations. These data add to the understanding of vitreoretinal attachment, which is important for a better comprehension of common vitreoretinal attachment pathologies.

## Introduction

Type VII collagen (Col VII) is renowned as the major component of anchoring fibrils (AF) [1,2]. AF are pliable, centrosymmetrically banded structures essential for the attachment of basement membranes to their underlying connective tissue matrix [2]. They are found as a network at the epithelial-mesenchymal border regions of various types of epithelia [1–11]. Because of the deformability of the anchoring fibrils, both the strain applied to the network by external forces, and its resistance, provided by entrapped fibrous stromal elements, might be minimized [12]. In the outer eye, AF have been demonstrated to perform an anchoring role beneath the basement membrane of the cornea, limbus, and conjunctiva [13–16]. In the inner eye, AF have never been described. Recently, however, Col VII expression was discovered in the inner human retina [17,18], and inner limiting membrane (ILM) of chicken [19]. These findings suggest a possible role for Col VII in vitreoretinal anchoring. The present study was undertaken to further characterize type VII collagen at the vitreoretinal interface. Our main purpose was to identify sites of Col VII expression in the normal human vitreoretinal interface. Any possible involvement of Col VII in vitreoretinal attachment should enhance our understanding of normal and pathological vitreoretinal adhesion mechanisms. Posterior vitreous detachment, for example, is a key factor involved in numerous ophthalmic pathologies, including the formation of macular holes, epiretinal membranes, rhegmatogenous retinal detachment, vitreoretinal traction syndromes, and other pathologies [20]. Furthermore, Col VII involvement would indicate that current enzymatic agents used for vitreoretinal separation might not target all necessary matrix components. Specific targeting should lead to less traumatic treatment: Current agents still result in adverse toxicity, lack of efficacy, or both [20]. We demonstrate that Col VII is an additional extracellular matrix component expressed at the vitreoretinal interface, possibly involved in vitreoretinal anchorage.

## Materials and Methods

Ethics Statement: Eyes were provided by the Euro Cornea Bank, Beverwijk (http://www.eurotissuebank.nl/comeabank/), the Netherlands. In the Netherlands, the usage of donor material is provided for by the Organ Donation Act (WOD: *Wet op de orgaandonatie*). In accordance with this law, donors provide written informed consent for donation, with an opt-out for the usage of leftover material for related scientific research. Specific requirements for the use in scientific research of leftover material originating from corneal grafting have been described in an additional document formulated by the Ministry of Health, Welfare, and Sport, and the BIS foundation (Eurotransplant; Leiden, July 21, 1995; 6714.ht). The current research was carried out in accordance with all requirements stated in the WOD and the relevant documents. Approval of the local medical ethics committee was not required, since the data were analyzed anonymously.

A total of 28 human eyes from 25 donors (11 men and 14 women), with ages varying between 17 and 82 years (mean donor age 65.8 years), were used (S1 Table). The eyes were without known ophthalmic disorders, although in two donors idiopathic epiretinal membranes were found during experiments. The eyes were analyzed immunohistochemically by means of light microscopy (LM), confocal microscopy, and transmission-electron microscopy (TEM). Homogenized retinal preparations were analyzed using Western blotting. For antibody specifications see S2 Table. Within 48 hours post mortem, all of the eyes were either fixed, or processed for Western blotting.

### Embedding in paraffin for light microscopy

Eyes were fixed by immersion in 2% paraformaldehyde (Polysciences Inc., Warrington, UK) in phosphate buffered saline (PBS) (pH 7.4). Two small holes were cut into the globe for better penetration of the fixative. By gently rotating the specimens during the washing, dehydration, and infiltration steps, the loss of vitreous was prevented. Ethanol (50–100%) and xylene were used as dehydrators, and paraffin embedding followed. Sections of 3–4 μm thickness were cut in the transverse (i.e., parallel to the longitudinal axis of the eye) and/or frontal planes (i.e., parallel to the equator of the eye) using a microtome (RM2265, Leica Microsystems, Heidelberg, Germany) and mounted on adhesive slides (Waldemar-Knittel, Braunschweig, Germany).

### Embedding in Technovit 8100 for light microscopy

Eyes were fixed for 1 hour by means of immersion in 2% paraformaldehyde, in 0.1 M phosphate buffer (pH 7.4). Again, two holes were made, and the eyes were gently rotated. The specimens were fixed additionally, for a total of 4 hours, in 2% paraformaldehyde. Specimens were washed in a solution of 6.8% sucrose in phosphate buffer overnight, and then briefly in distilled water before being dehydrated in acetone (30–100%). Specimens were pre-infiltrated, infiltrated, and embedded in Technovit 8100 (T8100) (Heraeus Kulzer, Wehrheim, Germany), according to the instructions of the manufacturer. Sections of 3 μm thickness were cut using a rotary microtome (Jung, Heidelberg, Germany) for LM.

### Immunohistochemical staining for LM: paraffin

Sections were deparaffinized with xylene followed by brief rehydration steps with ethanol (100–50%). After washing with deionized water, antigen retrieval was performed by incubating the sections with 0.01% type XXIV protease (Sigma, St. Louis, USA) in PBS for 30 minutes at room temperature. The sections were washed with PBS, and endogenous peroxidases were blocked by incubation in 0.1% H_2_O_2_ for 30 minutes. Next, sections were incubated in PBS with 2% bovine serum albumin (BSA) (Sanquin, Amsterdam, the Netherlands) and 5% serum for 10 minutes. Primary antibodies were diluted (1:500) in PBS with 1% BSA and added for 1 hour. The primary antibodies used were rabbit polyclonal anti-type VII collagen (Calbiochem, Darmstadt, Germany), mouse monoclonal anti-type VII collagen (LH7.2, Abcam, Cambridge, UK; clone 32, Chemicon, Temacula, California, USA), anti-glial fibrillary protein (GFAP) (an astrocyte marker, Sigma), and anti-vimentin (a Müller cell marker, Dako, Glostrup, Denmark) (S2 Table). After washing, secondary antibodies, conjugated with horseradish peroxidase, diluted (1:500) in PBS, 1% BSA, and 2% human serum were added for 1 hour at room temperature. These secondary antibodies included rabbit-anti-mouse peroxidase (RAMPO) and swine-anti-rabbit peroxidase (SARPO) (Dako). Sections were stained using 3-amino-9-ethylcarbazole (AEC, Sigma) and counterstained with hematoxylin. Some serial sections were counterstained using periodic acid-Schiff’s base according to a standard protocol.

### Immunohistochemical staining for LM: T8100

The T8100 sections from all four eyes were pretreated with 0.05% trypsin (Gibco, Paisley, Scotland) in Tris buffer (pH 7.8) containing 0.1% CaCl_2_, for 15 minutes at 37°C, for antigen retrieval. The sections were washed in phosphate buffer and incubated in 0.1 M citric acid pH 3.0 for 30 minutes at 37°C and washed. In order to block nonspecific binding of the primary antibodies, sections were incubated with PBS containing 2% BSA and 2% serum for 30 minutes at room temperature. The primary antibodies were diluted in phosphate buffer/1% BSA-c (Aurion, Wageningen, the Netherlands) (1:100). Sections were incubated in primary antibody initially for 2 hours at 37°C and then at room temperature overnight. Primary antibodies included the rabbit polyclonal and mouse monoclonals against Col VII. Sections were incubated in SARPO or RAMPO (1:500) in PBS with 1% BSA and 2% human serum for 1 hour. Sections were stained with 3-amino-9-ethylcarbazole and counterstained with hematoxylin. In control sections primary antibody was omitted.

#### Qualitative analysis of Col VII: light microscopy

Objective lens powers ranging from 20x to 100x were used for an analysis by LM of general and more detailed aspects of Col VII-labeled structures, and their relationship to retinal cells and layers.

#### Quantitative analysis of Col VII: light microscopy

Analysis of Col VII-labeled structures was done in different planes and included quantification, localization, and semi-quantitative size determination. Frontally cut sections were used for analyzing Col VII distribution at the equator in four quadrants (inferior-superior-temporal-nasal) by two authors (BW, RJW) independently, at 400x magnification. Sections of one eye did not contain sufficient retina tissue and were excluded. Numbers of Col VII-labeled spots were quantified per retinal layer. Transversally cut sections were used for analyzing distributions in anterior, equator, and posterior regions. The retinal sections were masked except for an open window at these regions, 3 mm in diameter. Two authors (LIL, RJW) analyzed the retina within these windows and quantified Col VII spots at 400x magnification.

#### Confocal microscopy

Sections from paraffin-embedded eyes and retinal whole mounts were used for double staining Col VII/GFAP experiments. In order to block nonspecific binding of the primary antibodies, sections were incubated with PBS containing 2% fatty acid free BSA (Sigma), for 30 minutes. The sections were incubated with primary antibodies (anti-Col VII rabbit polyclonal, 1:100; anti-GFAP mouse monoclonal, Sigma, 1:100) in 1%BSA/PBS, for 1 hour. Then, sections were incubated in rabbit anti-mouse tetramethyl rhodamine isothiocyanate (TRITC, Sigma, 1:500) and swine anti-rabbit fluorescein isothiocyanate (FITC, Dako, 1:500) for 45 minutes. Nuclei were visualized with (1:5000) 4’, 6’-diamino-2-phenylindole solution (DAPI, Sigma). All steps were performed at room temperature. Whole retina preparations were fixed (2% paraformaldehyde, 20 minutes), incubated in 1% Triton X-100/dH_2_O (10 minutes) and washed extensively to remove loose retinal pigment epithelia fragments. These preparations were then incubated in 3% H_2_O_2_/PBS for three days to reduce autofluorescence of the remaining pigment epithelium and 5%/PBS blocking buffer for 24 hours. Two primary antibody combinations were used, for increased probative value: (1) anti-Col VII rabbit polyclonal (1:200) and anti-GFAP mouse monoclonal (1:200), followed by the above mentioned secondary antibodies and DAPI, (2) anti-Col VII monoclonal (1:100) and anti-GFAP polyclonal (1:100), followed by the secondary antibodies donkey anti-mouse Red-X (Jackson, Suffolk, UK, 1:100) and donkey anti-rabbit FITC (Jackson, 1:100) and DAPI. All incubations were performed for 24 hours, all steps were performed at 4°C. In control samples, the primary antibodies were omitted. Visualization was done in a Leica TCS Sp2 confocal microscope, using Leica imaging software (LAS AF, LCS). The primary polyclonal anti-Col VII antibody specificity was tested on single-stained normal and Col VII deficient skin paraffin sections, which underwent the same procedures as described above.

#### Pre-embedding in epon

For ILM analysis, pieces of both posterior and anterior retina were dissected and fixed in 2% paraformaldehyde for 2 hours. These pieces were washed in 0.1% NaBH_4_ with 6.8% sucrose in phosphate buffer. Blocking was done overnight, using 5% BSA and 5% goat serum. Specimens were incubated in primary polyclonal antibody (1:100) overnight. They were washed and incubated in secondary gold-labeled antibody (Gold Colloid 2nm, British Biocell International, Cardiff, UK, 1:200) overnight. The samples were washed, fixed in 2% glutaraldehyde for 30 minutes, washed in 0.1 M cacodylate buffer, and contrasted using 1% osmium tetroxide. Then a silver enhancement step was performed (R-gent enhancer kit protocol, 1:1, Aurion) for 5 minutes, at room temperature. After washing, the samples were dehydrated in ethanol (50%-100%) and propylene oxide, and then mounted in epon. In retina control sections, the primary antibodies were omitted. The primary antibody specificity was tested on cornea samples which underwent the same procedure as described above. All steps were performed at 4°C. Sections of 100 nm were cut using an ultramicrotome (Ultracut type 701, Reichert-Jung, Austria) and placed on copper grids (150 mesh). Sections were incubated in uranyl acetate, methanol, and Pb/NaOH, respectively, for 2 minutes each. Analysis was done using a CM100 BioTwin TEM (Philips, Eindhoven, the Netherlands) and iTEM software (ResAlta Research Technologies, Golden, CO, USA).

#### Post-embedding in T8100

Sections of 100 nm were cut and mounted on grids. They underwent the same procedures as described for light microscopy. Then, after incubation of the primary polyclonal anti-Col VII antibody (1:100) for 1 hour at 37°C, samples were incubated in secondary gold-labeled antibody (Gold Colloid 5nm, British Biocell International, 1:200) for 1 hour at room temperature. Samples were fixed in 2% glutaraldehyde for 2 minutes after which silver enhancement (kit protocol 1:1) was performed for 10 minutes. Samples were contrasted by methylcellulose-uranyl acetate (9:1) for 15 minutes at 4°C and analyzed (CM100).

#### Western blot

Retinae were dissected and homogenized by pipetting for 10 minutes without any additives and divided into four groups (A, B, C, D). The supernatants were tested for collagenous content by establishing their susceptibility to digestion by bacterial type VII collagenase [21] (high purity grade, Sigma). In groups A and B, no collagenase was added. In groups C and D, collagenase was added to supernatants in 30 units/ml and 60 units/ml concentrations, respectively. Samples of groups B through D were incubated at 37°C for 120 minutes. Previously, the absence of nonspecific proteases in the collagenase batch had been confirmed [22]. Because the non-collagenous terminal domains of Col VII are known to be susceptible to pepsin, pepsin digestion was performed on fresh retinal supernatant for 1 hour at 37°C at a final concentration of 1 mg/ml. Polyacrylamide SDS electrophoresis was executed in conformity with Laemmli’s technique [23] as described [17], using 5% slab gels and a 72 mm wide 2D gel comb in a Bio-Rad Mini Protean II electrophoresis apparatus (Bio-Rad, Hercules, CA, USA). Afterwards, the gel was blotted to nitrocellulose paper using a Mini Protean II blotting unit (Bio-Rad) with 22 mM Tris, 0.05% SDS, 168 mM glycine, and 20% methanol as a transfer buffer. Then, the blot was blocked for 1 hour in TBS-buffer (20 mM Tris-HCl and 500 mM NaCl; pH 7.5) containing 3% BSA. Primary rabbit polyclonal antibody diluted 1:500 in TBS was added to the blot. After incubation overnight, the blot was washed with TBS containing 0.05% Tween-20 (TTBS). Secondary mouse-anti-rabbit antibody diluted 1:1000 in TBS was added. After 1 hour of incubation, the blot was washed with TTBS and incubated with alkaline phosphatase conjugated tertiary goat-anti-mouse antibody diluted 1:500 in TTBS for another hour. After washing with TTBS and alkaline phosphatase buffer (100 mM Tris-HCl, 100 mM NaCl, and 5 mM MgCl_2_, pH 9.5), the blot was developed with nitro blue tetrazolium and 5-bromo-4-chloro-3-indolyl phosphate in alkaline phosphatase buffer. Incubation and washing steps were performed at room temperature.

## Results

### Immunohistochemistry by light microscopy

#### Qualitative aspects of corpora amylacea

**Fig 1:**
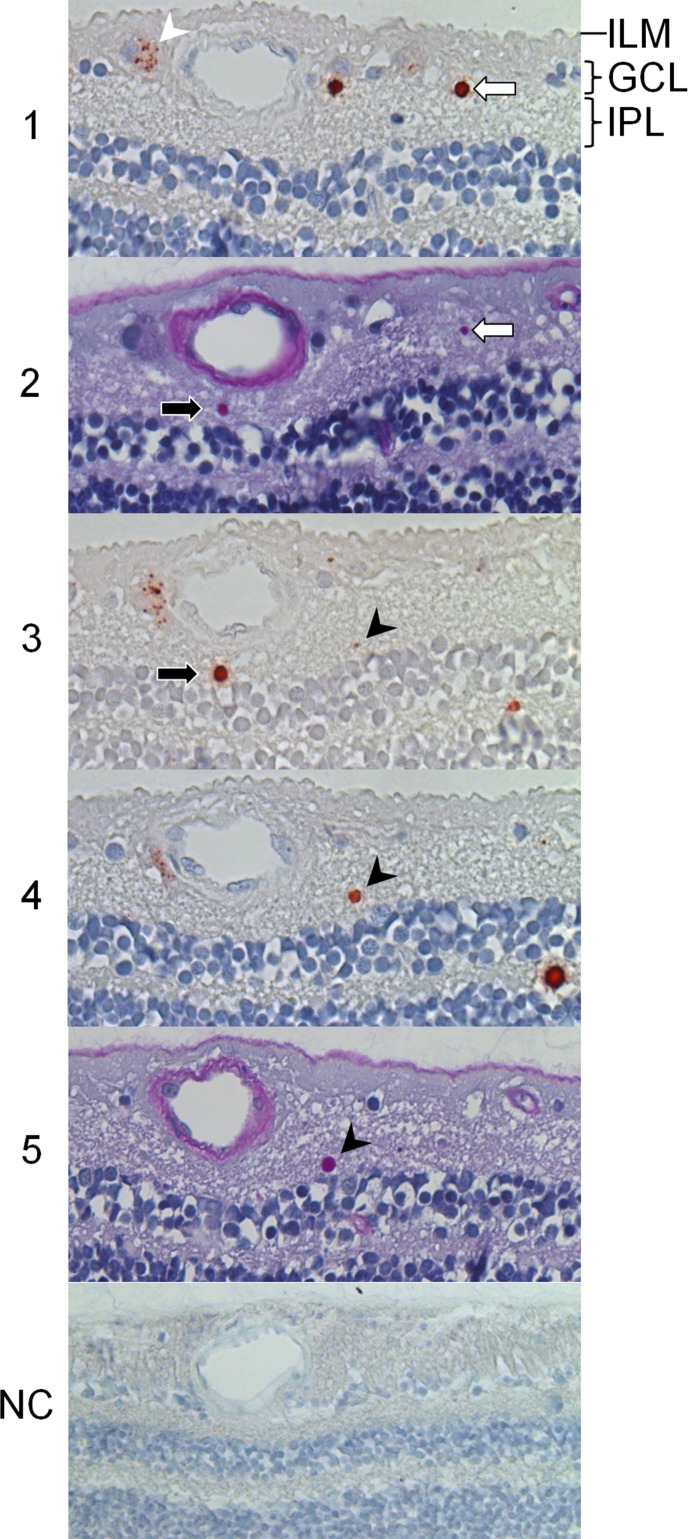
Retinal type VII collagen distribution. Immunohistochemical analysis at the vitreoretinal junction of a paraffin-embedded, serially sectioned (3–4 *μ*m) human donor eye evaluated by light microscopy. Anti-type VII collagen labeling with monoclonal LH7.2 (sections 1, 3 & 4) and periodic acid-Schiff (sections 2 & 5). Type VII collagen is present in astrocytic corpora amylacea. At least three corpora amylacea were sliced in this series, consecutively visualized in sections 1–2 (white arrows), sections 2–3 (black arrows) and sections 3–5 (black arrowheads). Corpora amylacea reside in the ganglion cell layer and inner plexiform layer (IPL). Type VII collagen is also present in small vesicles, clustered near large nuclei in the inner retina (white arrowhead). The vesicles reside outside the nucleus, and within the cytoplasm (sections 1, 2 & 3 compared). The cytoplasm of the type VII collagen yielding cell type seems more extensive than that of most other cells residing in the ganglion cell layer (GCL). Furthermore, their nuclei are larger and appear less dense in PAS (or hematoxylin) stains. The inner limiting membrane (ILM) does not label visibly for type VII collagen. Negative control section (NC) shows no labeling. (200 x)

By LM, anti-Col VII labeling was most obvious in cytoplasmic inclusions within astrocytic processes, known as corpora amylacea (CA). All CA were periodic acid-Schiff-positive (PAS) due to their glycoproteinaceous contents (Fig 1). The overlap between PAS- and Col VII-positivity seemed absolute. All anti-Col VII antibodies labeled CA similarly. CA were mainly located at the posterior pole. Their numbers diminished towards the pre-equatorial and anterior areas (Table 1). Some CA also reacted with anti-GFAP and anti-vimentin antibodies (S1 Fig). Negative control sections showed no labeling (Fig 1).

**Table 1:** Distribution of Col VII-labeled corpora amylacea.

		eye 1	eye 2	eye 3	eye 4	total	total %
**Age**		**29**	**56**	**70**	**74**		
**Gender**		**m**	**m**	**m**	**f**		
**Region**	anterior	**15**	**0**	**1**	**6**	**22**	**14.6**
	equator	**12**	**2**	**15**	**19**	**48**	**31.8**
	posterior	**29**	**3**	**14**	**35**	**81**	**53.6**
	***total***	***56***	***5***	***30***	***60***	***151***	***100***
**Layer**	internal limiting membrane	**0**	**0**	**0**	**1**	**1**	**0.7**
	nerve fiber layer	**2**	**0**	**0**	**4**	**6**	**4.0**
	ganglion cell layer	**3**	**2**	**0**	**17**	**22**	**14.6**
	inner plexiform layer	**44**	**3**	**26**	**35**	**108**	**71.5**
	inner nuclear layer	**7**	**0**	**4**	**3**	**14**	**9.3**
	outer plexiform layer	**0**	**0**	**0**	**0**	**0**	**0**
	outer nuclear layer	**0**	**0**	**0**	**0**	**0**	**0**
	**total**	***56***	***5***	***30***	***60***	***151***	***100*** #
**Size**	small	**39**	**1**	**18**	**43**	**101**	**66.9**
	medium	**15**	**4**	**12**	**13**	**44**	**29.1**
	large	**2**	**0**	**0**	**4**	**6**	**4.0**
	**total**	***56***	***5***	***30***	***60***	***151***	***100***

Numbers (n) of type VII collagen labeled corpora amylacea in human retina, as analyzed in transverse Technovit sections.

^#^Percentages rounded up to whole numbers. The most common corpus amylaceum is relatively small and located in the posterior retina in the inner plexiform layer. There are considerable inter-donor variations. No clear age-related characteristics are obvious.

#### Quantitative analysis of corpora amylacea

The quantitative analysis and distribution patterns were analyzed in transverse sections. The size of ganglion cell nuclei was used for semi-quantitative comparison of the CA. CA with the size of a ganglion cell nucleus were termed “medium” size. If they were obviously smaller than ganglion cell nuclei, they were termed “small.” If they were obviously larger than ganglion cell nuclei, they were termed “large.” In nearly two-thirds of the cases (67%), the CA were smaller than ganglion cell nuclei. Only a few were designated as “large,” measuring up to three times the size of a ganglion cell nucleus, mainly in the nerve fiber layer (data not shown). Overall, CA were mainly distributed in the inner plexiform layer (72%) and the posterior region (54%) (Table 1). Distribution of the (Col VII-labeled) CA appears to differ both inter-ocularly and intra-ocularly. For example, transverse sections of eyes “1,” “2,” and “3,” combined, contained about 96% of all the Col VII-labeled spots, whereas eye “1” only contained about 4%. Our sample size is too small to be able to indicate possible causes of these inter-individual differences. Therefore, the results have been arranged conveniently (Table 1).

#### Clustered small vesicles

**Fig 2:**
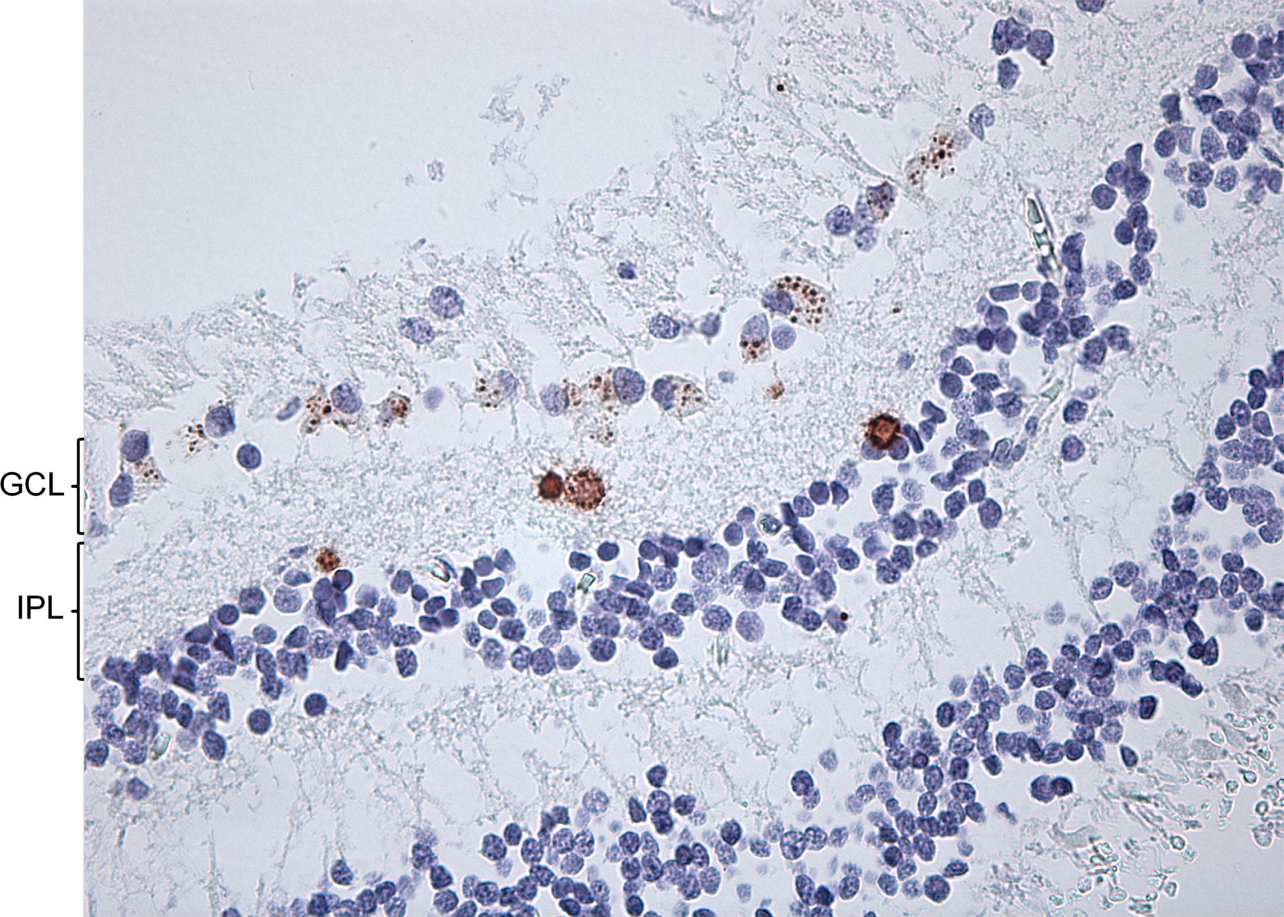
Immunohistochemical analysis at the vitreoretinal junction of a paraffin-embedded section. Anti-type VII collagen labeling with monoclonal LH7.2. This donor sample contains a lot of cells within the ganglion cell layer (GCL) that have Col VII-positive vesicles in their cytoplasm. Four corpora amylacea reside in the inner plexiform layer (IPL). (400x)

Col VII expression was observed in small, clustered vesicles within cells that resided in the ganglion cell layer and, to lesser extent, in the inner plexiform layer. In comparison to most ganglion cells, these vesicle-associated cells were shown to have large nuclei, with low nuclear hematoxylin staining densities (Fig 1). Furthermore, their PAS-stained cytoplasm appeared voluminous, in which the widely dispersed vesicles were probably confined to. Rarely, some non-dispersed vesicles were detected in the outer nuclear layer. These vesicles resided at (or close to) the cell nucleus (S2 Fig). The vesicles stained weakly with periodic acid-Schiff (Fig 1). They were not labeled by anti-vimentin antibodies (data not shown). In some donor samples, the vesicle-associated cells were numerous (Fig 2).

#### Inner limiting membrane

By LM, the donor eyes included in this study did not show conclusive Col VII expression at the ILM itself. In two donor eyes, an epiretinal membrane was observed, which did express detectable Col VII levels.

### Confocal microscopy

**Fig 3:**
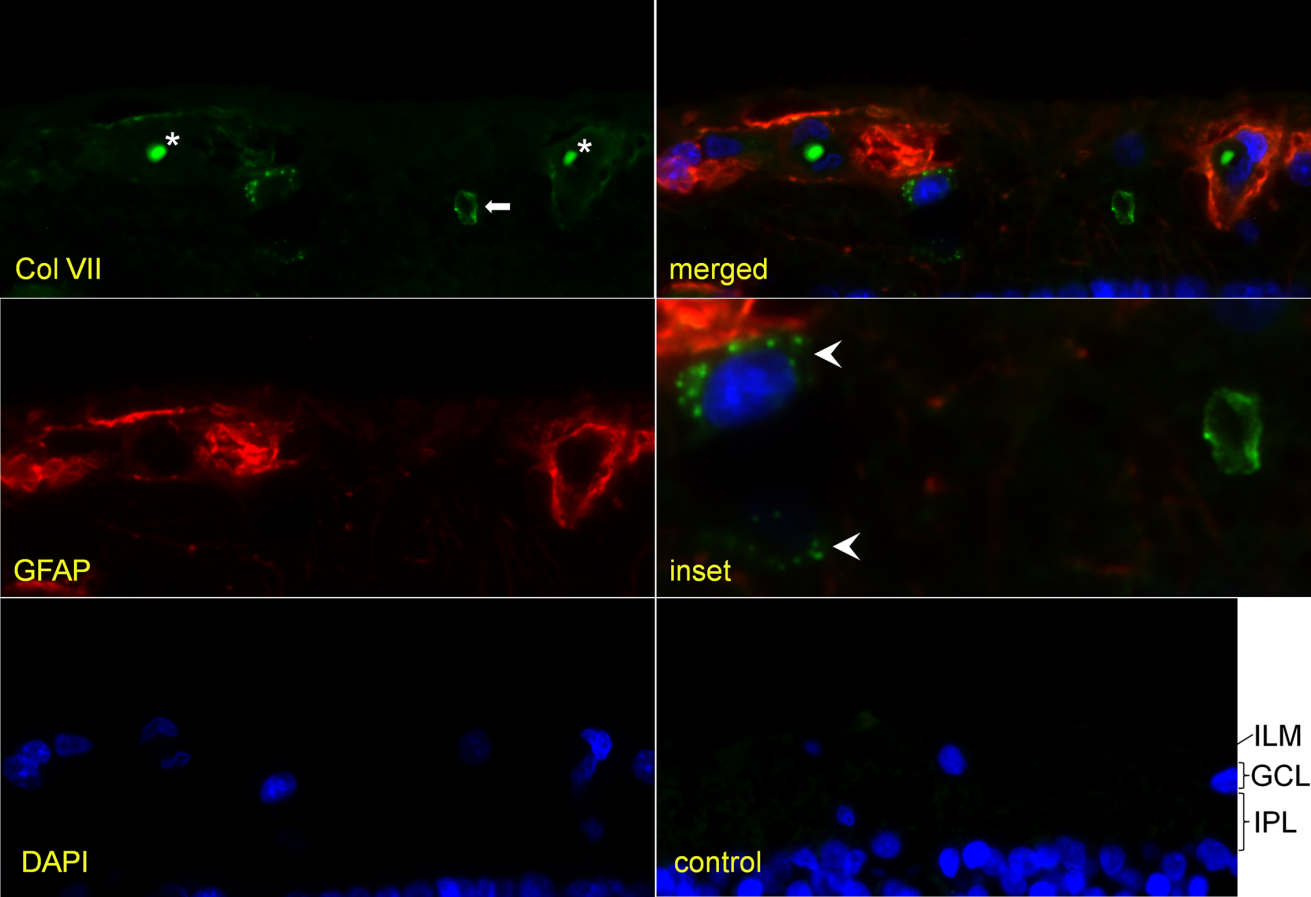
Confocal microscopy image of retinal section. Type VII collagen-positive vesicles (LH7.2; green) around two cell nuclei (white arrowheads) in the inner plexiform layer (IPL). Note filamentous GFAP (polyclonal; red) labeling within the nerve fiber layer and around blood vessels. Within the lumen of blood vessels, autofluorescent erythrocytes (*) are seen. They can be discriminated from the corpus amylaceum (white arrow) in terms of fluorescence intensity, size and location. The inner limiting membrane (ILM) directly above the left blood vessel shows some focal positivity for type VII collagen labeling. A control section does not show any Col VII or GFAP labeling. GCL ganglion cell layer. (400x, inset 960x)

**Fig 4:**
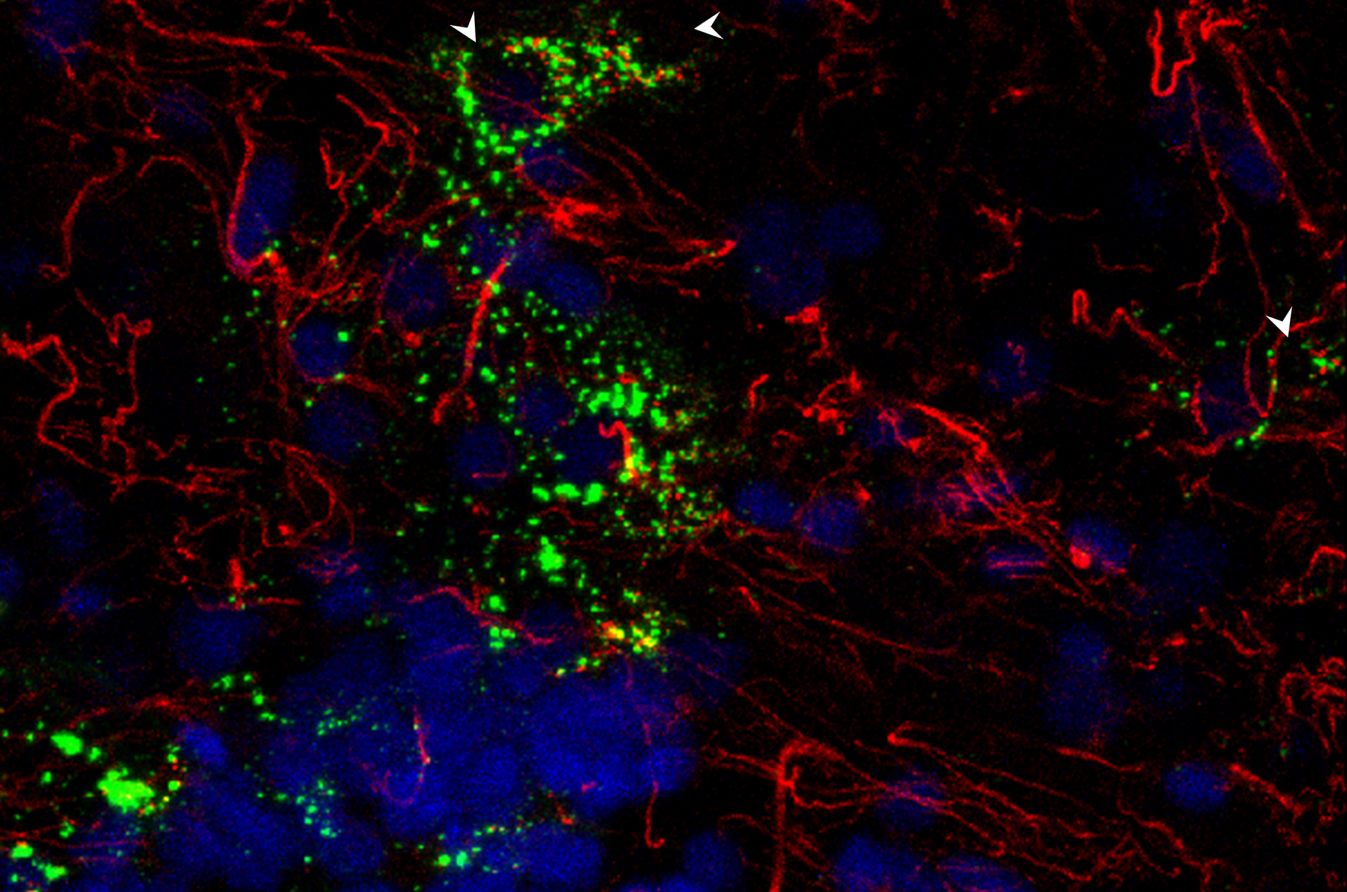
Confocal microscopy image of retinal wholemount. Type VII collagen-positive vesicles (LH7.2; green) colocalize with GFAP-positive vesicles (polyclonal; red) around cell nuclei (white arrowheads). In this donor sample, filamentous GFAP labeling is observed in the nerve fiber layer. This overlay projection is of the nerve fiber layer down to the inner IPL. Most Col VII vesicles are located at the junction of ganglion cell layer and inner plexiform layer. Although GFAP positive filaments meander around nuclei, the GFAP vesicles can be distinguished from sectioned filaments. (630x)

**Fig 5:**
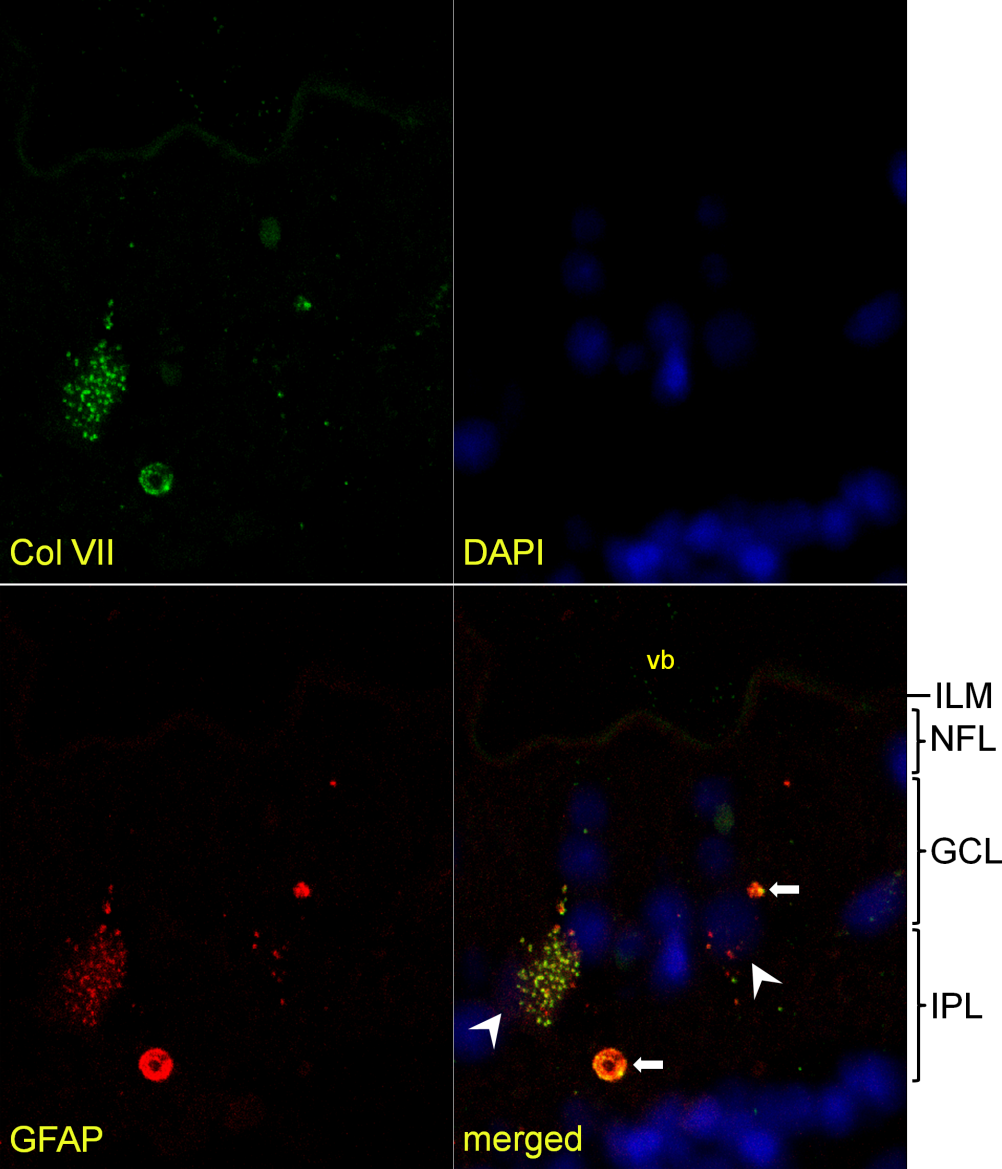
Colocalization of type VII collagen and GFAP within vesicle clusters and corpora amylacea. Image of the inner retina, paraffin section. Type VII collagen labeling (polyclonal; green) colocalizes with that of GFAP (monoclonal; red). The vesicle cluster around one nucleus (left white arrowhead) contains both Col-VII and GFAP-positive vesicles, while another cluster (right white arrowhead) has few Col VII-positive vesicles. Most vesicles contain both type VII collagen and GFAP and label yellowish, but others contain either Col-VII or GFAP. In this donor sample, no filamentous GFAP labeling is detected (by monoclonal antibodies) at the nerve fiber layer (NFL). Two corpora amylacea can be seen, both labeled dually (white arrows). ILM inner limiting membrane, GCL ganglion cell layer, IPL inner plexiform layer, vb vitreous body. (400x)

On confocal microscopy, Col VII-positive vesicles were intensely labeled and located within the cytoplasm, at variable distances from the cell nucleus, and without apparent polarization (Fig 3). The vesicle-associated cells resided mainly in the ganglion cell layer and inner plexiform layer (S1 Video). In most donor samples, Col VII and GFAP-positive vesicles colocalized within clusters besides filamentous labeling of the nerve fiber layer (Fig 4). Filamentous labeling was increased around blood vessels (Fig 3). Some donor samples lacked GFAP-positivity in the Col VII-positive vesicle clusters (Fig 3). Others had GFAP/Col-VII-positive CA and vesicles, but lacked filamentous labeling (Fig 5) Retinal control sections showed no labeling (Fig 3). Skin sections served as positive (normal skin) and negative control (rEBD skin). The primary polyclonal antibody specifically stained the dermal-epidermal basement membrane from a healthy donor sample, in contrast to that of a Col VII deficient donor sample (S4 Fig).

### Transmission electron microscopy

**Fig 6:**
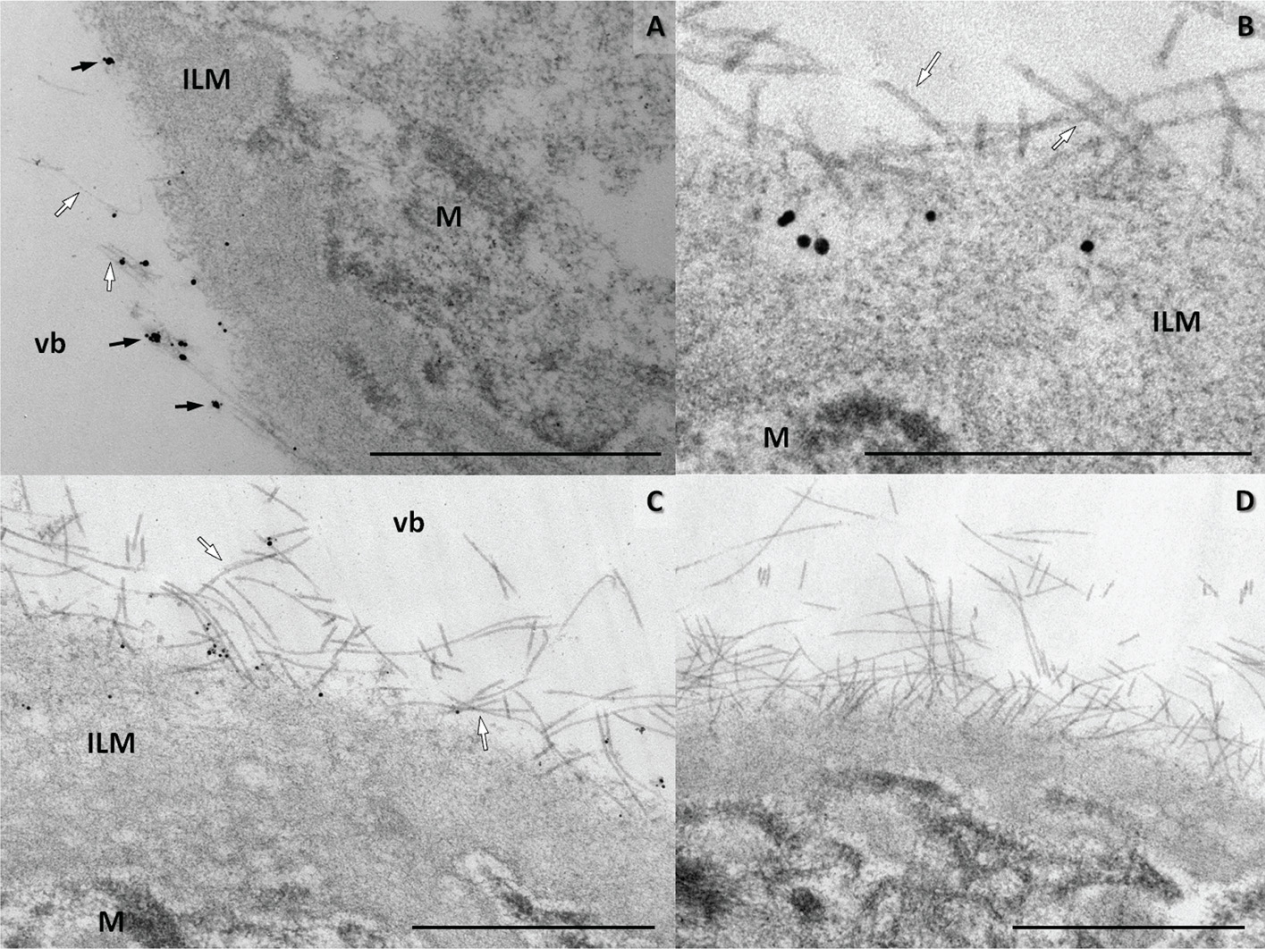
Type VII collagen is visualized at the vitreoretinal interface by immuno-TEM (epon). Immuno-electron microscopic image of the inner limiting membrane of a human retina. (**A-C**) Immunogold-labeled polyclonal antibody directed against type VII collagen (black arrows) is found in the direct vicinity of vitreous fibrils (white arrows) at the vitreoretinal interface. Some gold labeling is also situated within the inner limiting membrane (ILM). (**A**) Anterior retina, showing sparse, clustered labeling. Vitreous collagen fibrils join the retina. Bar 1 μm. (**B**) The posterior retina has frequent gold labeling at a depth of 150–200 nm in the inner limiting membrane. Bar 500 nm. (**C**) Posterior retina, gold labeling for type VII collagen is mostly at the retinal side of the vitreous fibrils. Müller cell endfeet (M) remain unlabeled. vb vitreous body. (**D**) Negative control of posterior retina shows no labeling. Bar 1μm.

**Fig 7:**
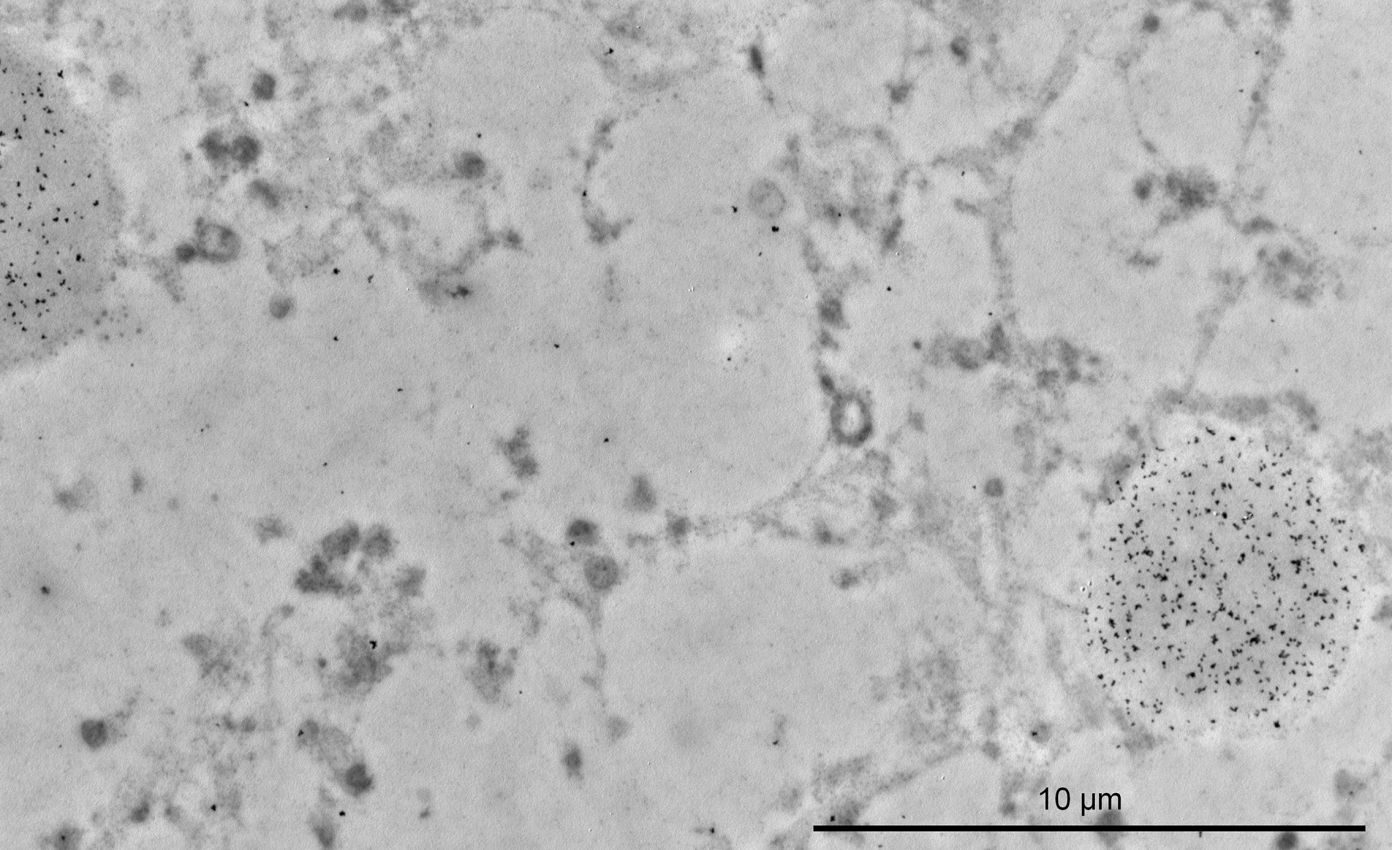
Type VII collagen is visualized within corpora amylacea (T8100). Immuno-electron microscopic image of the inner plexiform layer of a human retina. Immunogold-labeled polyclonal antibody directed against type VII collagen labels two corpora amylacea diffusely. Within the corpora amylacea, there is no evidence of fibrillar collagen or banding. Some background labeling is present. Bar 10 μm.

Col VII was detected in both posterior and anterior retinal ILM, with good specificity, in T8100 and epon embedded samples. The ILM contained gold labeling not only on the vitreous side (Fig 6A–6C), but also in deeper parts of the ILM itself. Mostly, concomitant deep and superficial gold labeling was observed (Fig 6A and 6C). Gold labeling was also located alongside numerous vitreous fibers. There seemed to be no increase in gold labeling in the trajectory underneath their insertions in the ILM. There was no obvious variation in gold labeling toward the posterior pole, despite the increase in ILM thickness. Classical arcade shaped AF were not distinguished. Positive control cornea samples showed gold labels on AF at the basement membrane zone. The gold labeling was intense and very specific, when using the polyclonal antibody (S5 Fig). Both the Müller cell endfeet and the negative controls showed no labeling (Fig 6D), but had some background staining from silver enhancement. In the T8100 specimens, CA contained diffusely dispersed immunogold labeling (Fig 7). In contrast to the corneal basement membrane, no fibrillar shapes could be distinguished within CA. Furthermore, the vesicles (and the associated cells) could not be identified, due to absent gold labeling at their appropriate locations.

### Western blot

**Fig 8:**
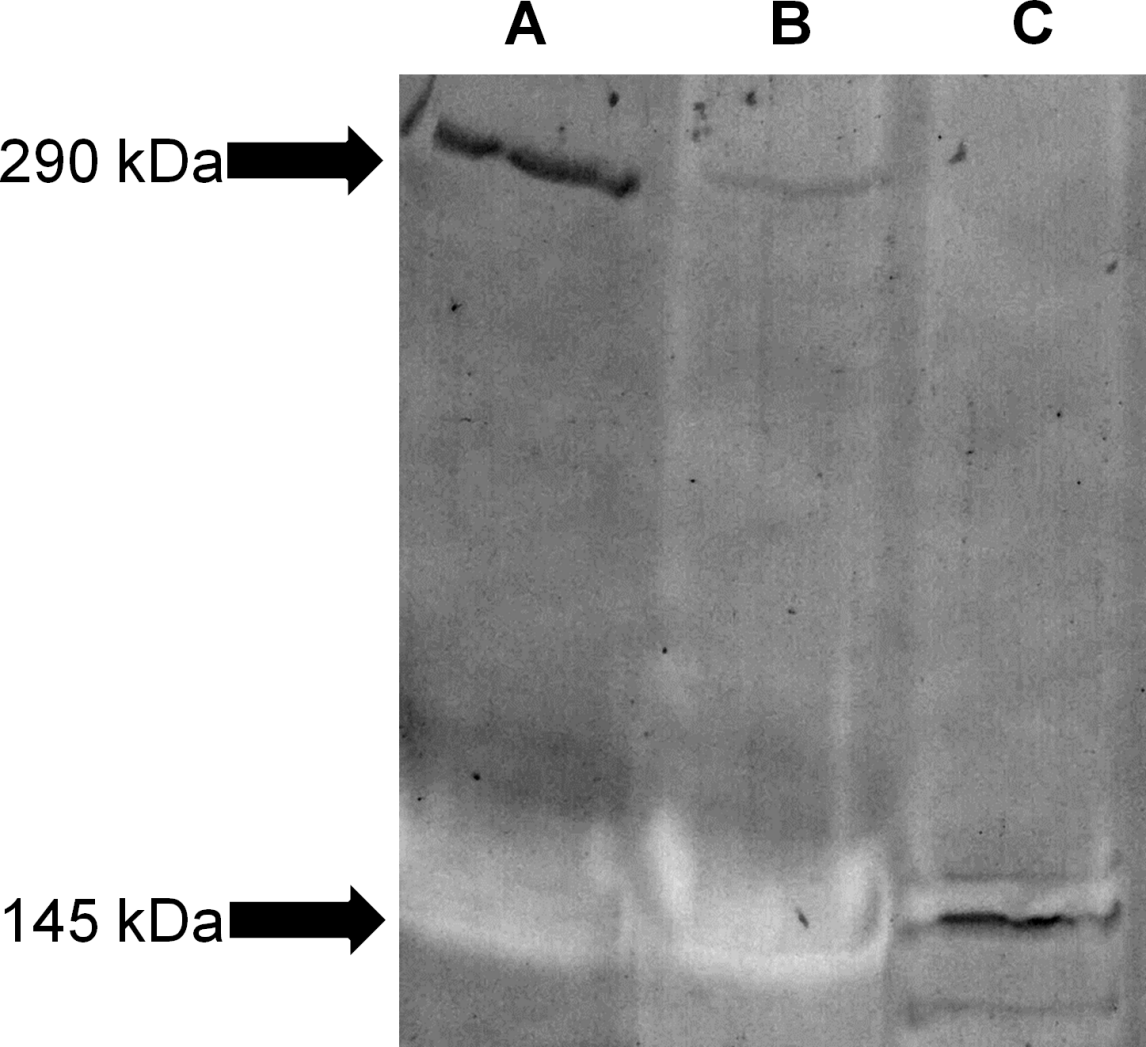
Western blot confirms type VII collagen content in retinal substrates. Western blot stained for type VII collagen. A 290 kDa band appeared in samples (directly) placed in reducing agent buffer (group/lane **A**). Omitting the use of reducing agents resulted in fading of the 290 kDa band (group/lane **B**). When samples were incubated in 30 units/ml collagenase, the 290 kDa band disappeared completely, while a 145 kDa band appeared (group/lane **C**).

The immunoblotting showed a specific band at 290 kDa (Fig 8A). After two hours of incubation in collagenase (30 units/ml), the 290 kDa band disappeared completely due to digestion. Meanwhile, a 145 kDa band of degradation products was formed (Fig 8C). Without collagenase addition, the 290 kDa band faded in two hours (Fig 8B) due to auto-digestion. After two hours of incubation in collagenase (60 units/ml), all bands had disappeared (group D; data not shown). The use of an LH7.2 monoclonal antibody resulted in the appearance of a 320 kDa band in addition to the 290 kDa and 145 kDa bands (S6 Fig). The 320 kDa band probably corresponds to type VII procollagen. After pepsin digestion, all bands had disappeared (S7 Fig).

## Discussion

### Corpora amylacea

The Col VII positive “spots” previously described by Ponsioen et al. [17], were found to correspond to CA. CA are smooth (or granular) structures that occur in round or oval forms, measuring 1 to 50 μm in diameter [24–27]. Ocular CA are found in the inner retinal layers (i.e. nerve fiber layer through inner nuclear layer) and optic nerve [27–29]. Here, they reside most frequently in astrocytes, or occasionally within neuritic processes, but also exist as extracellular deposits. They are thought to be of mitochondrial origin [30]. Since astrocytes, and their processes, are closely associated with retinal ganglion cells [28,31] and contain GFAP [32] and vimentin [33], our qualitative and quantitative CA characteristics correlate with earlier investigations [28,34]. We expect that during normal ageing (or neurodegeneration) some Col VII molecules (or epitopes) lose their function and are therefore phagocytized by astrocytes and stored in CA. In correspondence to similar protein accumulations within CA [35], this would benefit retinal neuroprotection. Otherwise, obsolete proteins could aggravate the immune system, leading to neuronal damage [33,36,37].

### Vesicles

The Col VII-labeled vesicles, which Ponsioen termed “dots” [17], reside in a cell type with decreased nuclear density. Low densities may arise when the nuclear euchromatin is increased, indicating active gene transcription. Since the Col VII-labeled vesicles were also labeled by anti-GFAP antibodies during CA quantification experiments in paraffin, we suspected these Col VII/GFAP-labeled vesicles to reside in activated astrocytes. Additionally, the distribution of these vesicle-associated cells is comparable with that of astrocytes. Both predominantly reside in the nerve fiber layer and ganglion cell layer [32,38], although astrocytes can migrate to other layers where vesicles were found, e.g. the inner plexiform layer and outer nuclear layer. Alternatively, ganglion cells are a candidate cell type, based on the predominant ganglion cell layer distribution of the vesicle-associated cells. The presence of Col VII in idiopathic epiretinal membranes would be consistent with either cell type, since both astrocytes and ganglion cell neurites can be present in these membranes [39,40]. The Col VII positive vesicles might reflect Col VII synthesis, phagocytosis, or both. At present, our data is not robust enough to differentiate between these possibilities.

### Vitreoretinal interface

Immunogold labeling suggests low concentrations of Col VII in comparison to other collagens, as in skin [41]. Small quantities of Col VII may be clinically relevant, since absence or malfunction of Col VII leads to severe blistering in the skin. The vitreoretinal Col VII distribution suggests interactions with vitreous collagen fibers, therefore probably performing a role in vitreoretinal attachment. We visualized the NC1 domains of Col VII inside the ILM, although AF were not seen. *In vivo* [11,14,15,42–44] and *in vitro* [6,12] investigations acknowledge difficulties in visualizing AF, despite apparent Col VII labeling. Some authors have advocated that only one end of the skin AF inserts into the basement membrane [45], while others state that lateral aggregation might not be necessary for a functional role [46]. In skin, laterally aggregated AF can entrap stromal matrix components by looping back into the lamina densa [1,8,9,47,48]. It is conceivable that the lateral aggregation is not compulsory for a functional role. Furthermore, in rat trachea, the AF that are actually entrapping underlying fibers are few, which would indicate that their arcade shape might not provide the sole anchoring modus [46]. Generally, the dermal AF density seems to correlate with the frictional exposure of the tissue, suggesting a function in stabilizing the epithelial-stromal border [2]. Despite this important function, Col VII constitutes only 0.001% of the total skin tissue collagens [41]. In the inner eye, we expect this proportion and thus the number of AF (if present) to be even lower, given the challenge of adequate immunolabeling. Moreover, AF numbers vary widely both among individuals and within the same subject [49]. Furthermore, epitope recognition by anti-Col VII antibodies can be influenced, even in cell cultures, as illustrated by a study using different fixation techniques [1]. For these reasons and concomitant technical difficulties [2], demonstrating Col VII or visualizing AF can be a challenge in itself [46].

### Limitations

Our study demonstrates vitreoretinal Col VII depositions, without the obvious AF formations found at dermal and corneal basement membrane zones [2,14]. The lack of evident AF can be considered as a limitation of our study, although a low AF density and alternative functional Col VII formations [50] would sufficiently explain this. Our dataset is too small to draw conclusions about age-related influences, such as the numbers of CA containing Col VII, or the weakening of the vitreoretinal interface because of degenerating adhesive molecules that could give rise to age-related vitreous degeneration and posterior vitreous detachment. In contrast to whole mounts and paraffin samples, no vesicles were labeled in the T8100 and epon samples. The glycol methacrylate T8100 was elected for maintaining good tissue morphology, but at the possible expense of labeling sensitivity. Firstly, glycol methacrylate cannot be removed completely. Therefore, slides are etched in order to retrieve antigens. Secondly, because of its hydrophobic nature, residual T8100 can hinder antigen-antibody complex formation. In epon samples, the lack of vesicular labeling could be explained by limited antibody penetration.

## Conclusion

We detected Col VII expression 1.) in corpora amylacea, 2.) within intracellular vesicles, of an undisclosed cell type residing in the ganglion cell layer and inner plexiform layer, and 3.) at the inner limiting membrane. At these sites, no anchoring fibrils could be visualized.

## Supporting Information

S1 Fig Retinal vimentin distribution. Immunohistochemical analysis at the vitreoretinal junction of a paraffin-embedded section. Monoclonal anti-vimentin labeling is seen in Müller cells, but also in corpora amylacea of the inner plexiform layer (IPL) (white arrows). GCL ganglion cell layer. (20x)
(TIF)

S2 Fig Retinal type VII collagen distribution: inner plexiform layer vs. outer nuclear layer. Immunohistochemical analysis at the vitreoretinal junction of a paraffin-embedded, serially sectioned human donor eye evaluated by LM peroxidase staining. Polyclonal anti-type VII collagen labeling is seen in close vicinity to the nucleus in the outer nuclear layer (arrowhead), while reactivity is seen dispersed in the cytoplasm in the nerve fiber layer (arrow). ILM inner limiting membrane, NFL nerve fiber layer, GCL ganglion cell layer, IPL inner plexiform layer, INL inner nuclear layer, OPL outer plexiform layer, ONL outer nuclear layer PR photoreceptors, vb vitreous body. Bar 500 nm.
(TIF)

S3 Fig Type VII collagen expression in (human) epiretinal membrane. (**A**) Immunohistochemical staining of a 73-year-old female retina, polyclonal anti-collagen type VII on paraffin sections. A dense epiretinal membrane is seen, as a dark blue layer containing cells. Between the epiretinal membrane and the ILM, type VII collagen labeling is seen (red). Labeling seems to be strongest directly underneath the cells. T8100 section of a 77-year-old male. (**B**) Hematoxylin, light microscopy. (**C-E**) Electron microscopy (post-embedded, polyclonal anti-Col VII antibody). (**C**) Overview of E. (**D**) Gold labels are found within the epiretinal membrane. Arcing fibers reminiscent of anchoring fibrils associate with the gold-labeled antibodies. (**E**) Intense gold labeling is seen without apparent arced fibers on lower magnification.
(TIF)

S4 Fig Antibody specificity test: immunofluorescence staining intensities in skin of healthy control and recessive Epidermolysis Bullosa Dystrofica (rDEB) patient. Arrows point to type VII collagen deposition at the basement membrane zone. In contrast to the healthy control, the rDEB patient’s type VII collagen deficient skin shows no immunofluorescence when tested with the polyclonal antibody. Please note that the remaining fluorescence in the rDEB is due autofluorescence of the stratum corneum. Bars 100 μm.
(TIF)

S5 Fig Human Bowman’s membrane of cornea, immunogold-labeled for type VII collagen (T8100). Immuno-electron microscopic image. Polyclonal antibody directed against NC-1 domain of type VII collagen. (**A**) Intense immunogold-labeled lamina densa. Because of the arced shapes of the type VII collagen dimers, their NC-1 domains reside in the lamina densa as well as the superficial Bowman’s layer. The intermediate lamina fibroreticularis itself, where the collageneous parts of the type VII collagen molecule reside, therefore has little gold labeling. An epithelial cell is seen above the basement membrane (top); the stroma is seen beneath the basement membrane (bottom). Bar 10 μm. (**B**) Inset. The lamina densa is intensely gold-labeled. (C) Schematic diagram of epithelial anchorage to the stroma (Reprinted from: *Soma T*, *Nishida K*, *Yamato M*, *et al*. *Histological evaluation of mechanical epithelial separation in epithelial laser in situ keratomileusis*. *J Cataract Refract Surg*. *2009;35(7) 1251–1259*. Copyright 2009, with permission of Elsevier). Note the location of the NC-1 globuli of type VII collagen in C, depicted as black ovals, corresponding to the gold labels in **A** and **B**.
(TIF)

S6 Fig Western blot of retinal substrates. Western blot of human (23-year-old female and 64-year-old male, pooled) retinal substrate. Monoclonal primary antibody (LH7.2). Both the 290 kDa and 145 kDa bands appear, although the latter only faintly. Control remained negative. [1] *Isolation of collagens*, *see*: *Van Deemter M*, *Pas H*, *Kuijer R*, *Van der Worp RJ*, *Hooymans JM*, *Los LI*. *Enzymatic breakdown of type II collagen in the human vitreous*. *Invest Ophthalmol Vis Sci*. *2009;50(10)*:*4552–4560*.
(TIF)

S7 Fig Western blot of retinal substrates; pepsin digestion. Western blot of human (23-year-old female and 64-year-old male, pooled) retinal substrate. Monoclonal primary antibody (LH7.2). After one hour of digestion at 37°C with pepsin at a final concentration of 1 mg/ml (**P+**), no bands appeared, in contrast to omitting pepsin addition (**P-**). [1] Isolation of collagens, see: *Van Deemter M*, *Pas H*, *Kuijer R*, *Van der Worp RJ*, *Hooymans JM*, *Los LI (2009) Enzymatic breakdown of type II collagen in the human vitreous*. *IOVS 50*: *4552–4560*.
(TIF)

S1 Table Donor materials. Twenty-eight donor eyes without known ophthalmic disorders were used for our experiments. Experiment type and donor characteristics are listed.
(XLSX)

S2 Table Antibodies. Overview of antibody characteristics.
(XLSX)

S1 Video Confocal microscopy imaging session presented as video. Retinal whole mount, from outer part of nerve fiber layer through inner part of inner plexiform layer. The polyclonal anti-type VII collagen antibody (green) labels intracellular vesicles. Vesicles within the same clusters may label with monoclonal anti-GFAP antibodies (red), indicating co-localization of both proteins within one vesicle or co-occurrence of both types of vesicles within one cell type. In the nerve fiber layer (first 2 seconds) GFAP labels glial filaments in a horizontal plane. The filaments then meander deeper into the retina. The GFAP positive vesicles can be distinguished from transversely ‘cut’ filaments. Cell nuclei are blue (DAPI).
(AVI)
