# Supplementary figures and images for: Type VII Collagen Expression in the Human Vitreoretinal Interface, Corpora Amylacea and Inner Retinal Layers
Source: PLoS One. 2015 Dec 28;10(12):e0145502. doi: 10.1371/journal.pone.0145502 (PMC4692387; doi:10.1371/journal.pone.0145502)

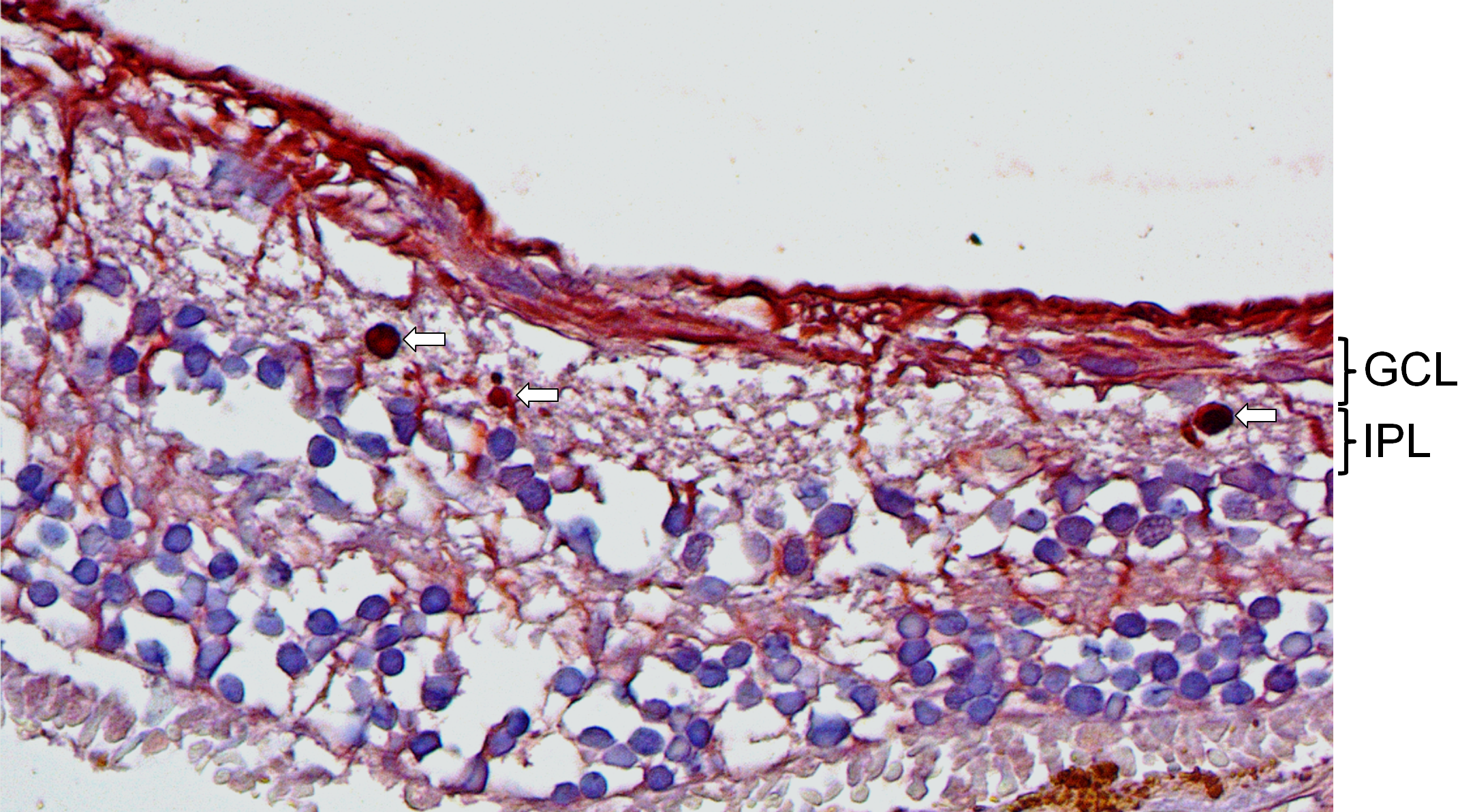

Supplement: S1 Fig — Immunohistochemical analysis at the vitreoretinal junction of a paraffin-embedded section. Monoclonal anti-vimentin labeling is seen in Müller cells, but also in corpora amylacea of the inner plexiform layer (IPL) (white arrows). GCL ganglion cell layer. (20x) (TIF) [file pone.0145502.s001.tif]

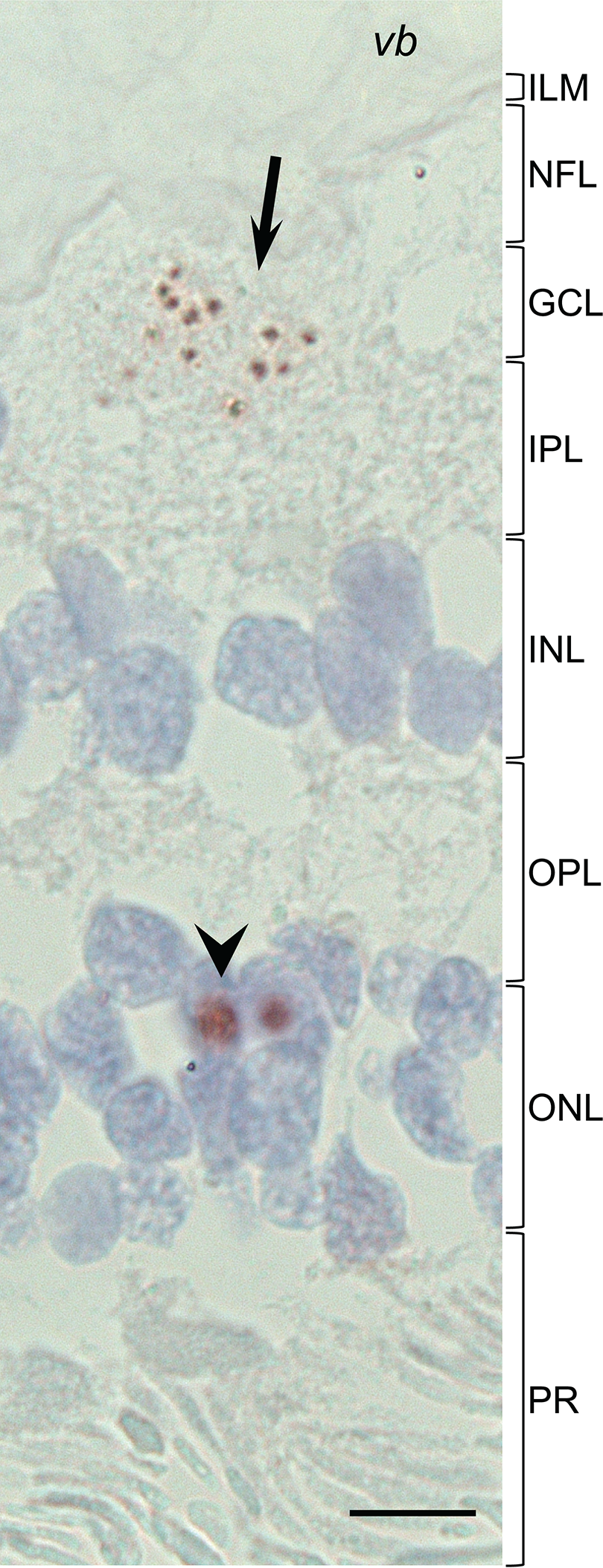

Supplement: S2 Fig — Immunohistochemical analysis at the vitreoretinal junction of a paraffin-embedded, serially sectioned human donor eye evaluated by LM peroxidase staining. Polyclonal anti-type VII collagen labeling is seen in close vicinity to the nucleus in the outer nuclear layer (arrowhead), while reactivity is seen dispersed in the cytoplasm in the nerve fiber layer (arrow). ILM inner limiting membrane, NFL nerve fiber layer, GCL ganglion cell layer, IPL inner plexiform layer, INL inner nuclear layer, OPL outer plexiform layer, ONL outer nuclear layer PR photoreceptors, vb vitreous body. Bar 500 nm. (TIF) [file pone.0145502.s002.tif]

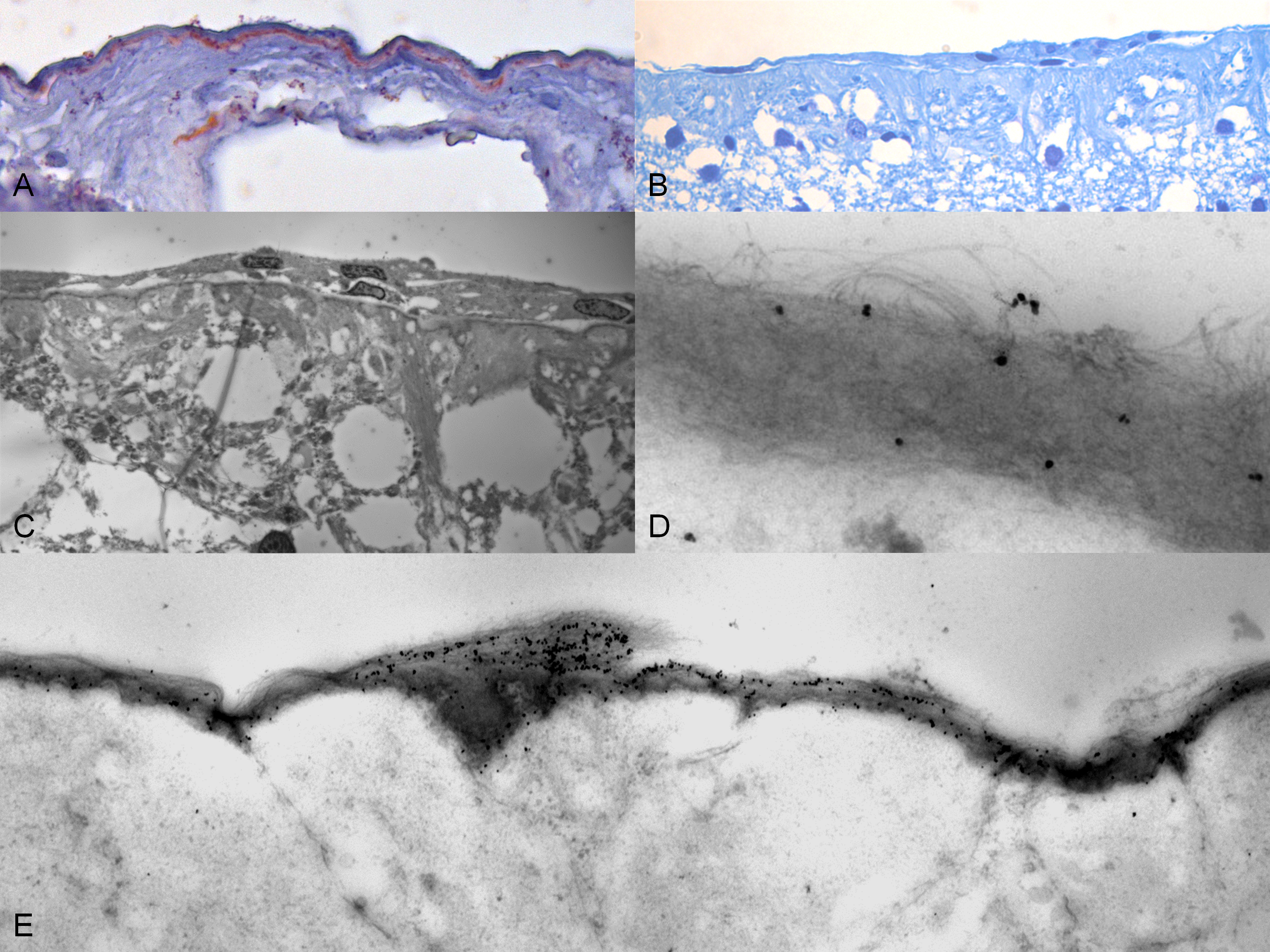

Supplement: S3 Fig — (A) Immunohistochemical staining of a 73-year-old female retina, polyclonal anti-collagen type VII on paraffin sections. A dense epiretinal membrane is seen, as a dark blue layer containing cells. Between the epiretinal membrane and the ILM, type VII collagen labeling is seen (red). Labeling seems to be strongest directly underneath the cells. T8100 section of a 77-year-old male. (B) Hematoxylin, light microscopy. (C-E) Electron microscopy (post-embedded, polyclonal anti-Col VII antibody). (C) Overview of E. (D) Gold labels are found within the epiretinal membrane. Arcing fibers reminiscent of anchoring fibrils associate with the gold-labeled antibodies. (E) Intense gold labeling is seen without apparent arced fibers on lower magnification. (TIF) [file pone.0145502.s003.tif]

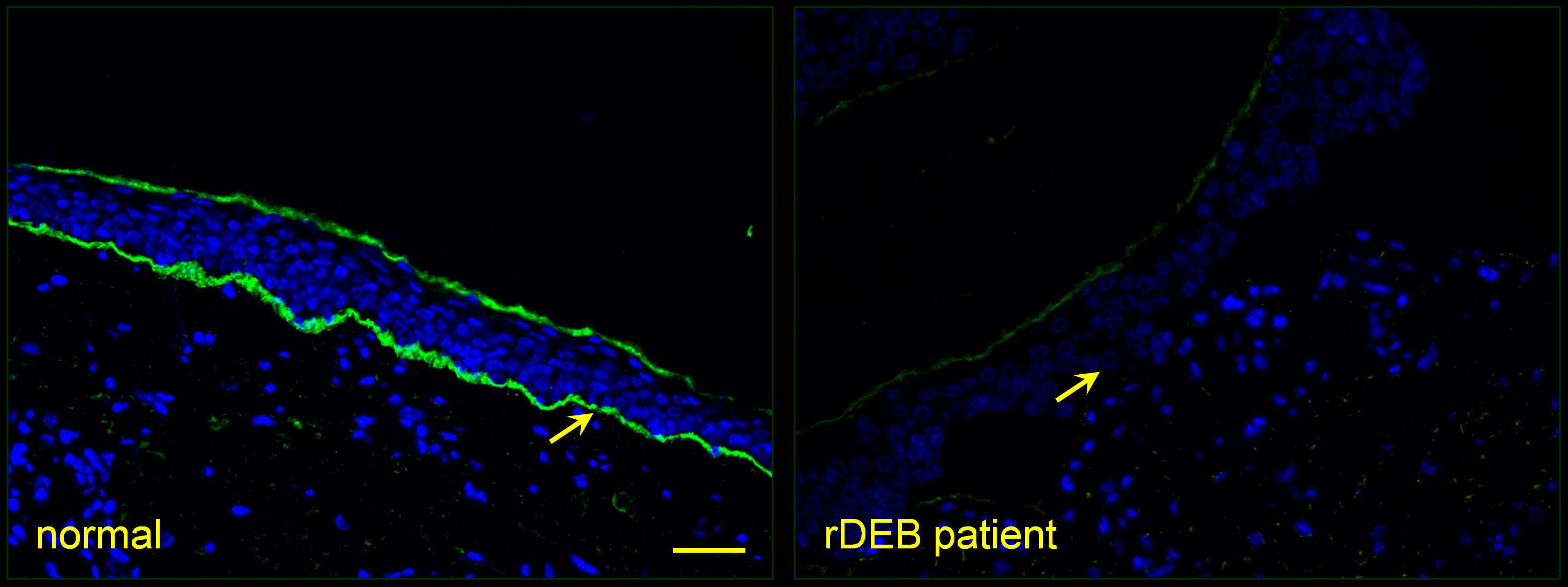

Supplement: S4 Fig — Arrows point to type VII collagen deposition at the basement membrane zone. In contrast to the healthy control, the rDEB patient’s type VII collagen deficient skin shows no immunofluorescence when tested with the polyclonal antibody. Please note that the remaining fluorescence in the rDEB is due autofluorescence of the stratum corneum. Bars 100 μm. (TIF) [file pone.0145502.s004.tif]

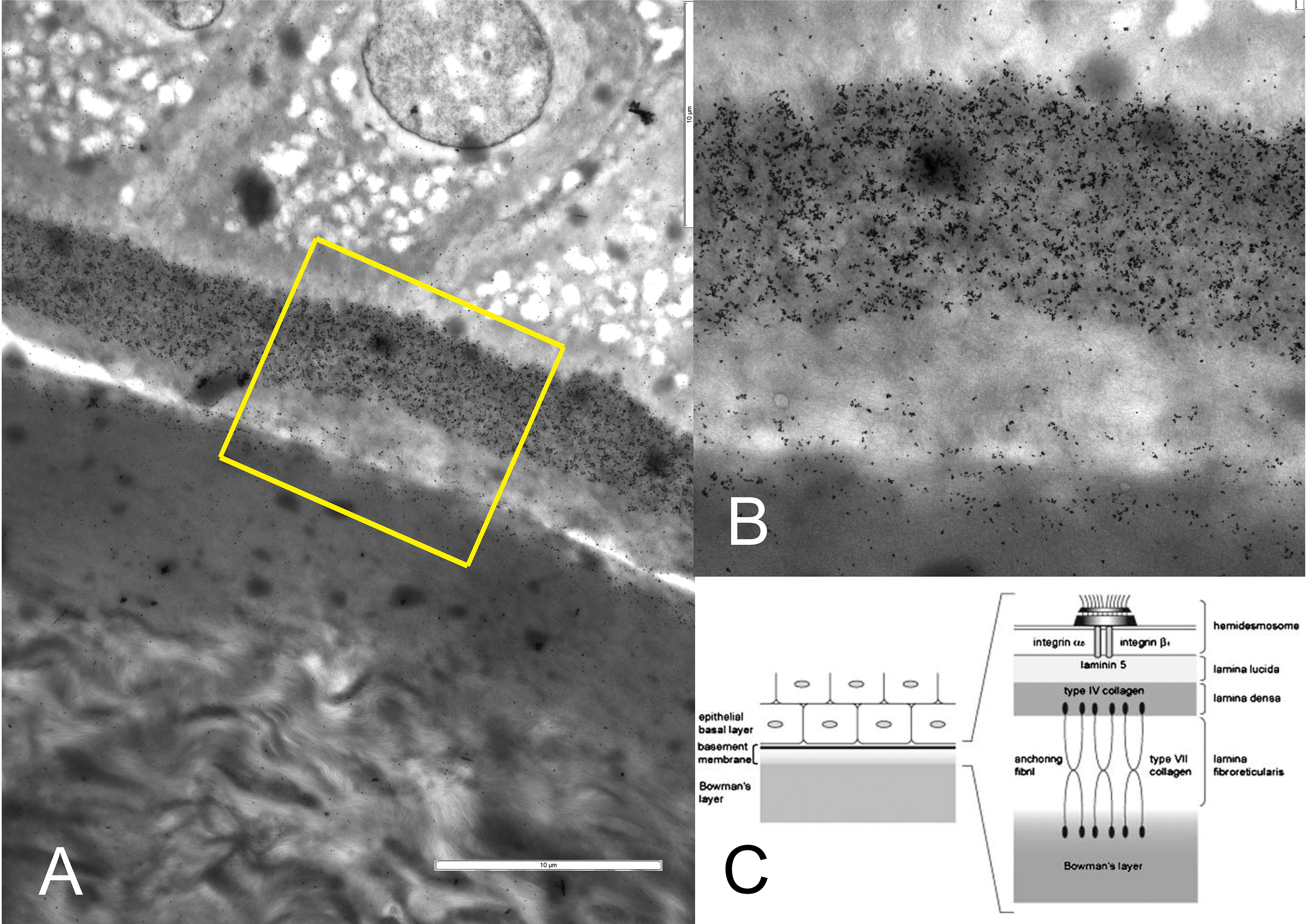

Supplement: S5 Fig — Immuno-electron microscopic image. Polyclonal antibody directed against NC-1 domain of type VII collagen. (A) Intense immunogold-labeled lamina densa. Because of the arced shapes of the type VII collagen dimers, their NC-1 domains reside in the lamina densa as well as the superficial Bowman’s layer. The intermediate lamina fibroreticularis itself, where the collageneous parts of the type VII collagen molecule reside, therefore has little gold labeling. An epithelial cell is seen above the basement membrane (top); the stroma is seen beneath the basement membrane (bottom). Bar 10 μm. (B) Inset. The lamina densa is intensely gold-labeled. (C) Schematic diagram of epithelial anchorage to the stroma (Reprinted from: Soma T, Nishida K, Yamato M, et al. Histological evaluation of mechanical epithelial separation in epithelial laser in situ keratomileusis. J Cataract Refract Surg. 2009;35(7) 1251–1259. Copyright 2009, with permission of Elsevier). Note the location of the NC-1 globuli of type VII collagen in C, depicted as black ovals, corresponding to the gold labels in A and B. (TIF) [file pone.0145502.s005.tif]

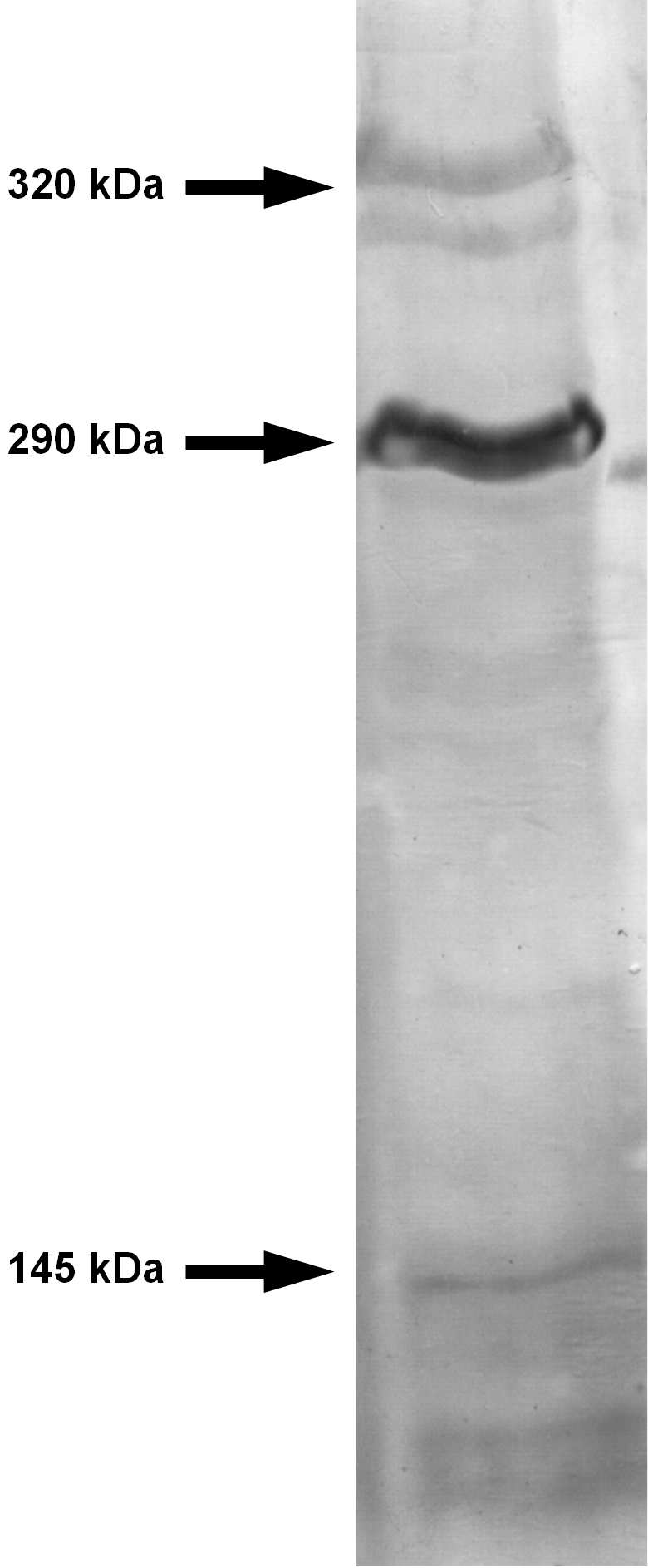

Supplement: S6 Fig — Western blot of human (23-year-old female and 64-year-old male, pooled) retinal substrate. Monoclonal primary antibody (LH7.2). Both the 290 kDa and 145 kDa bands appear, although the latter only faintly. Control remained negative. [1] Isolation of collagens, see: Van Deemter M, Pas H, Kuijer R, Van der Worp RJ, Hooymans JM, Los LI. Enzymatic breakdown of type II collagen in the human vitreous. Invest Ophthalmol Vis Sci. 2009;50(10):4552–4560. (TIF) [file pone.0145502.s006.tif]

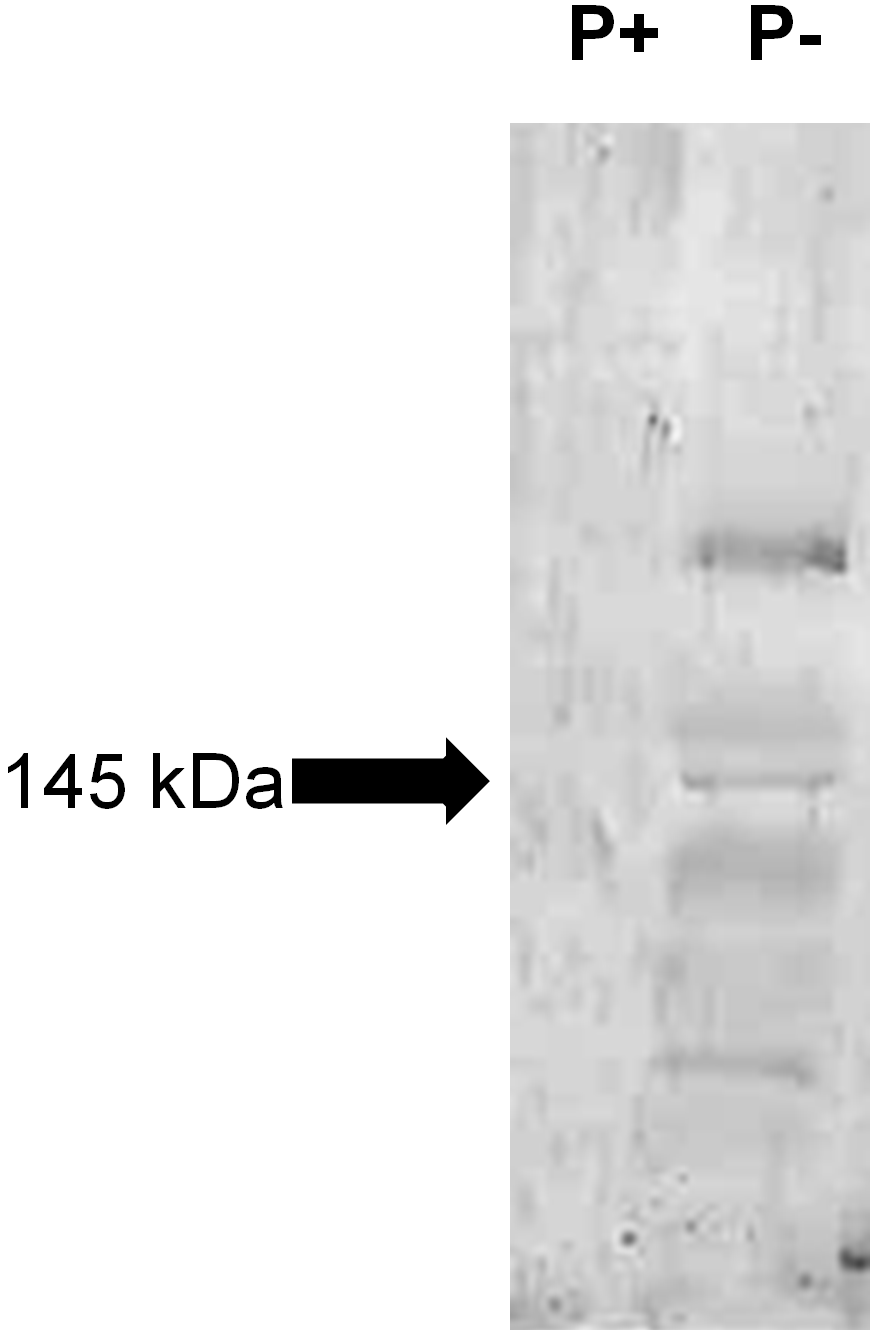

Supplement: S7 Fig — Western blot of human (23-year-old female and 64-year-old male, pooled) retinal substrate. Monoclonal primary antibody (LH7.2). After one hour of digestion at 37°C with pepsin at a final concentration of 1 mg/ml (P+), no bands appeared, in contrast to omitting pepsin addition (P-). [1] Isolation of collagens, see: Van Deemter M, Pas H, Kuijer R, Van der Worp RJ, Hooymans JM, Los LI (2009) Enzymatic breakdown of type II collagen in the human vitreous. IOVS 50: 4552–4560. (TIF) [file pone.0145502.s007.tif]
